# Supplementary material for: LIGHT (TNFSF14) enhances osteogenesis of human bone marrow-derived mesenchymal stem cells
Source: PLoS One. 2021 Feb 19;16(2):e0247368. doi: 10.1371/journal.pone.0247368 (PMC7895395; doi:10.1371/journal.pone.0247368)
Supplement: S1 Fig — (A) Negative marker (CD34, CD45, and CD19) staining in BM-MSCs. (B) Positive marker (CD90, CD44, and CD105) staining in BM-MSCs. (C) LTβR expression on the hBM-MSC surface. Each marker’s expression level was determined using FACS analysis. Filled histogram represents the isotype control (mouse IgG); open histogram represents each antigen. (DOCX) [file pone.0247368.s001.docx]

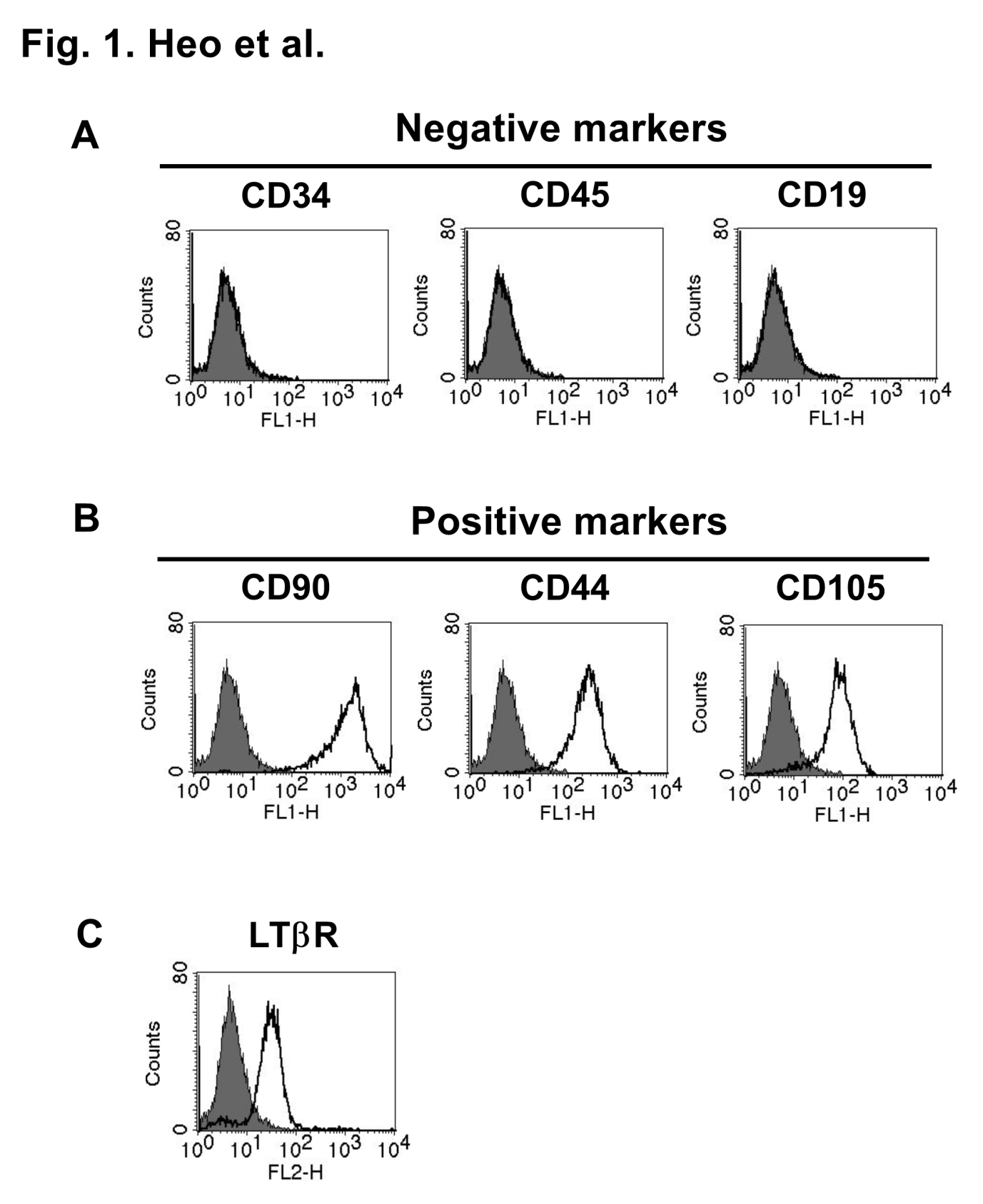


**S1 Fig. Quality test on hBM-MSCs.**

(A) Negative marker (CD34, CD45, and CD19) staining in BM-MSCs. (B) Positive marker (CD90, CD44, and CD105) staining in BM-MSCs. (C) LTβR expression on the hBM-MSC surface. Each marker’s expression level was determined using FACS analysis. Filled histogram represents the isotype control (mouse IgG); open histogram represents each antigen.
