# Supplementary material for: LIGHT (TNFSF14) enhances osteogenesis of human bone marrow-derived mesenchymal stem cells
Source: PLoS One. 2021 Feb 19;16(2):e0247368. doi: 10.1371/journal.pone.0247368 (PMC7895395; doi:10.1371/journal.pone.0247368)

Supplementary Figure 2.
Original western blots used for Fig. 3B

ALP/Day 0, 18

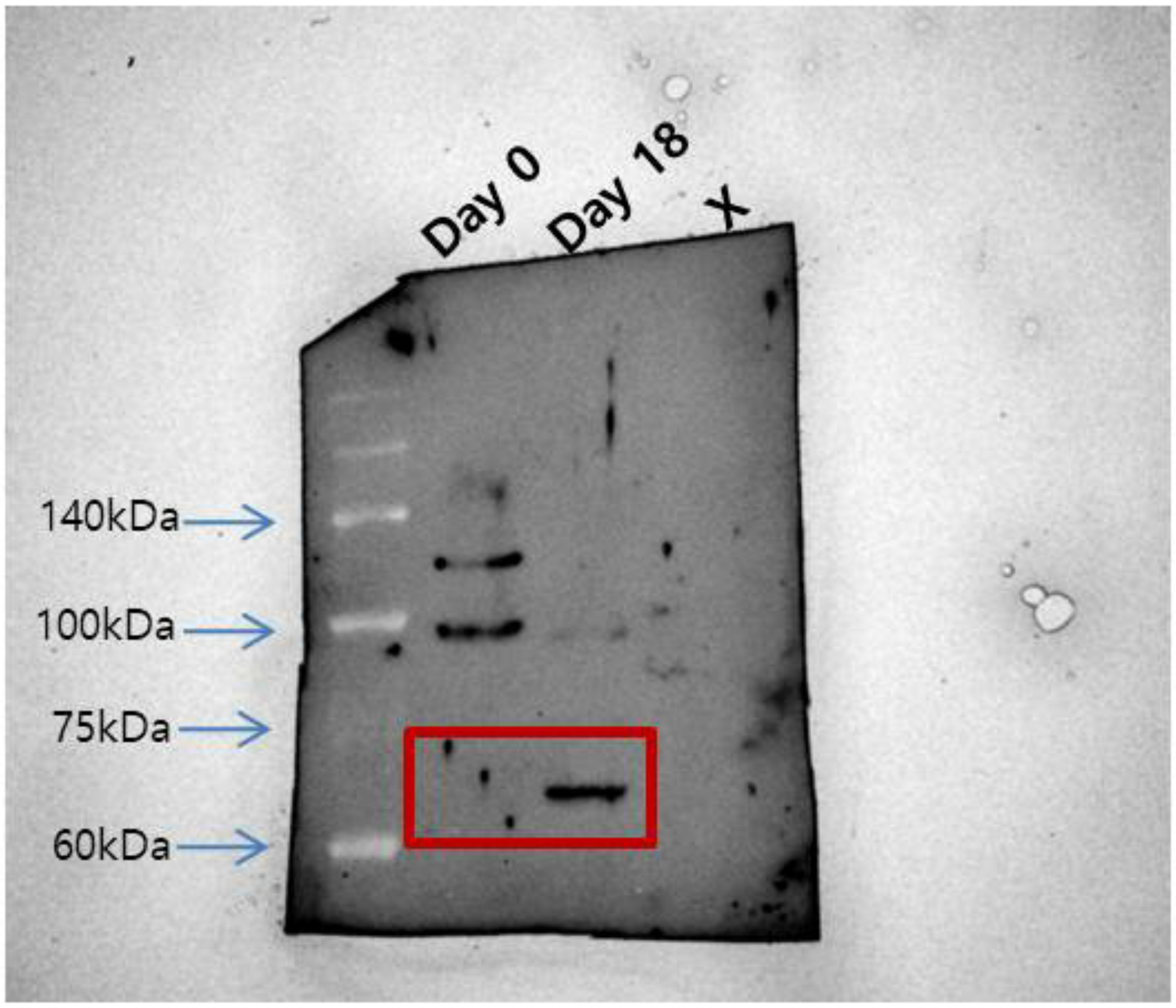

COL1A1/Day 0, 18

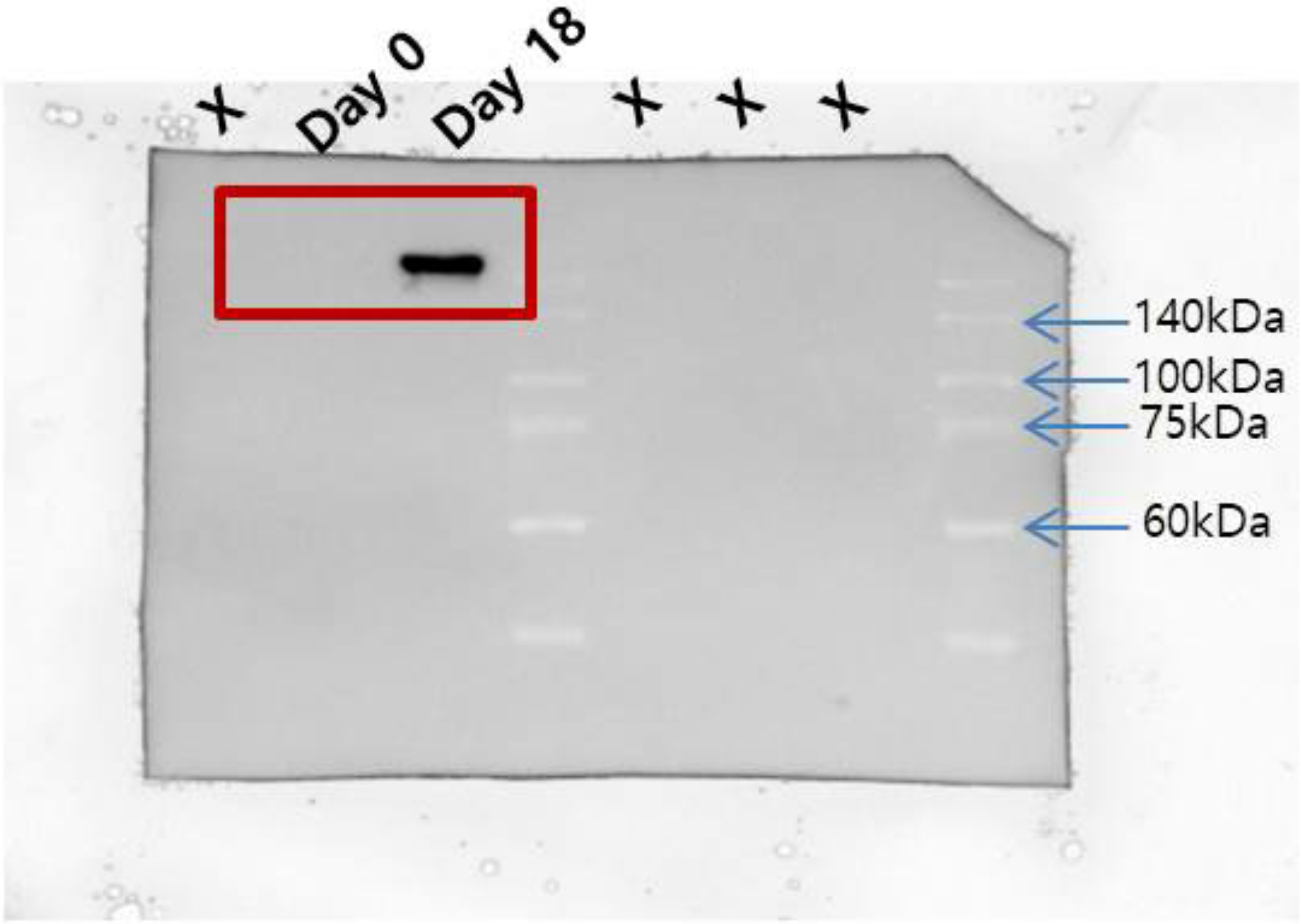

OSX/Day 0, 18

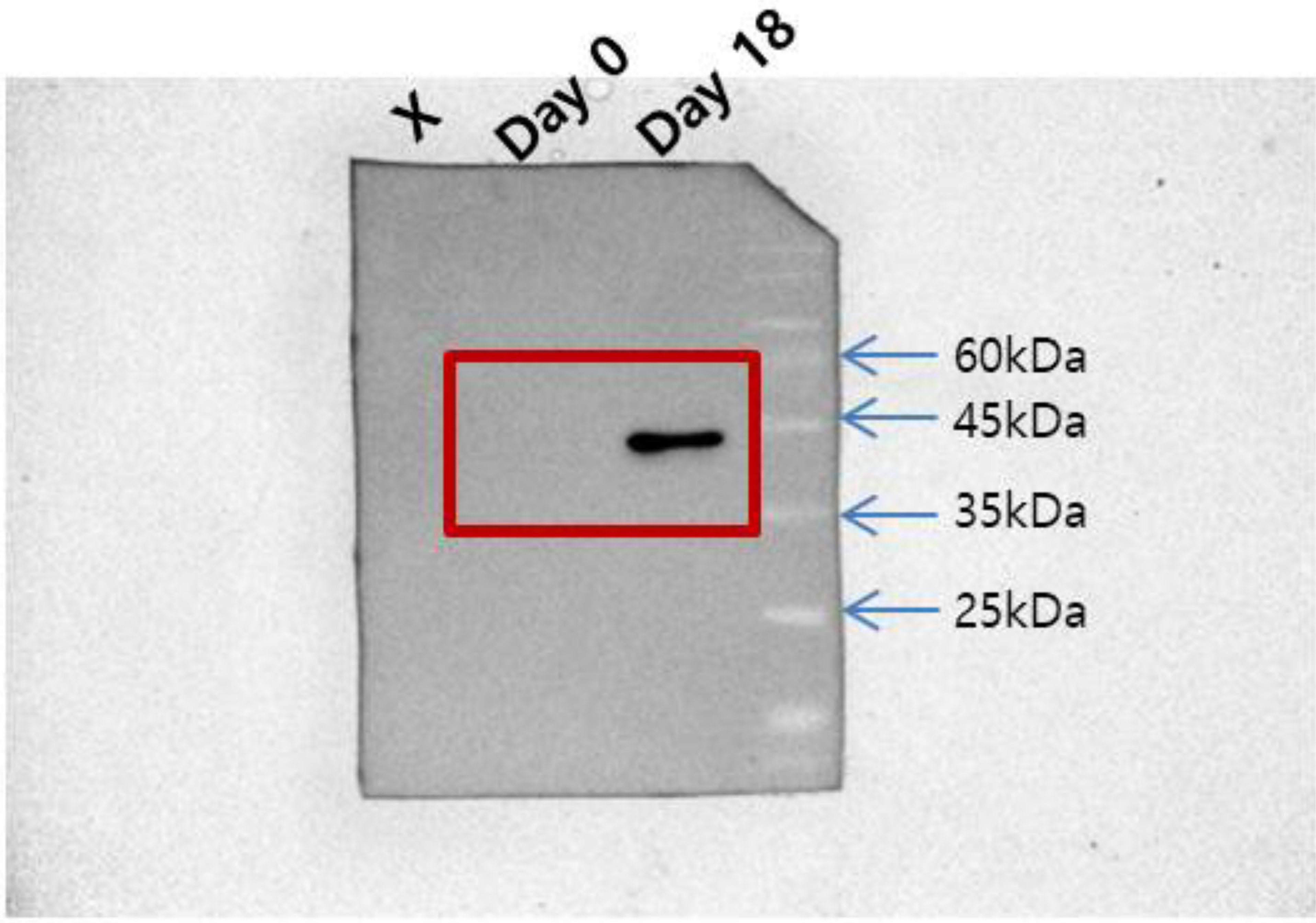

β-actin/Day 0, 18

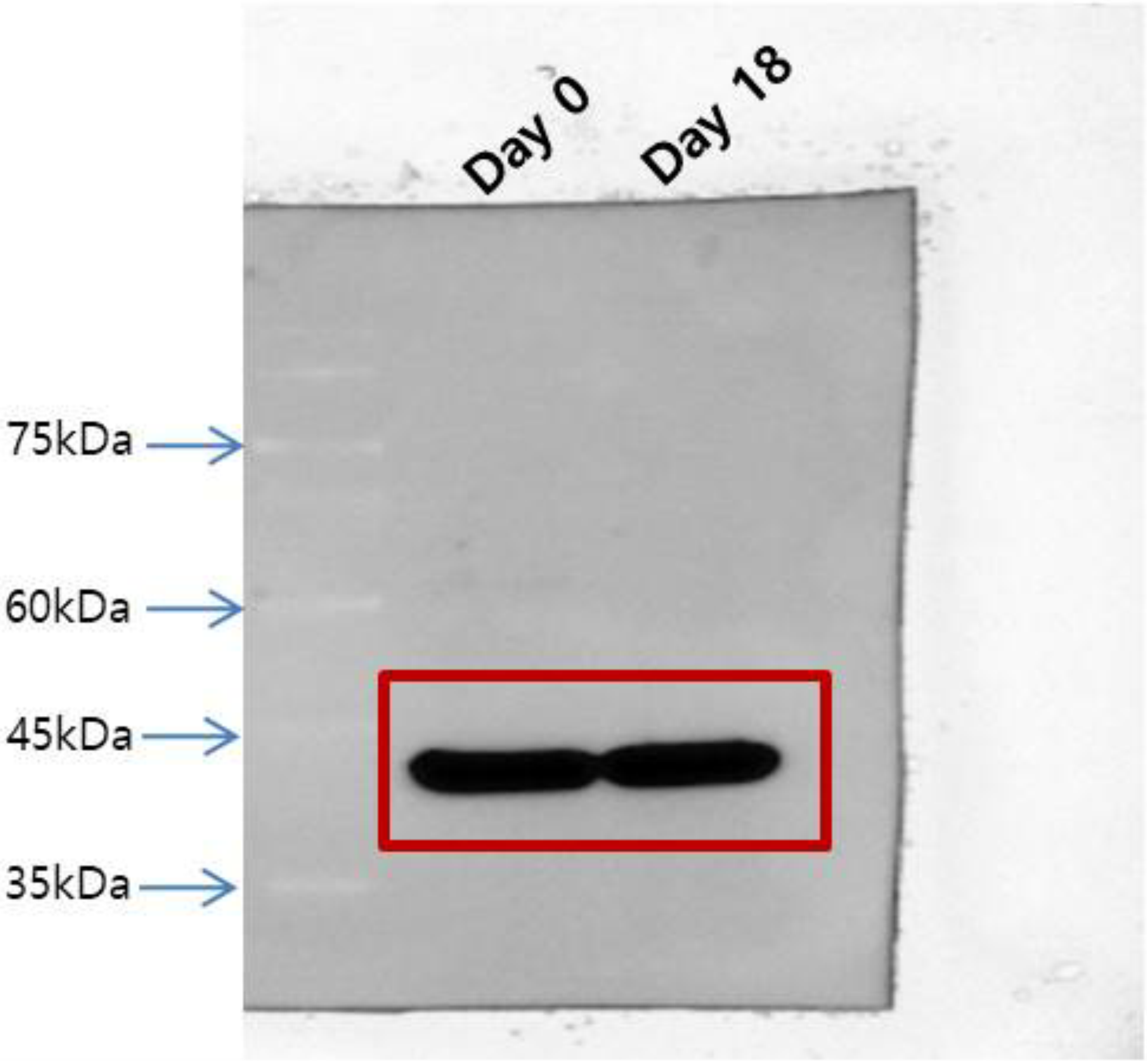

Supplementary Figure 2. continued

Original western blots used for Fig. 3B

ALP/rhLIGHT 0, 50ng/ml

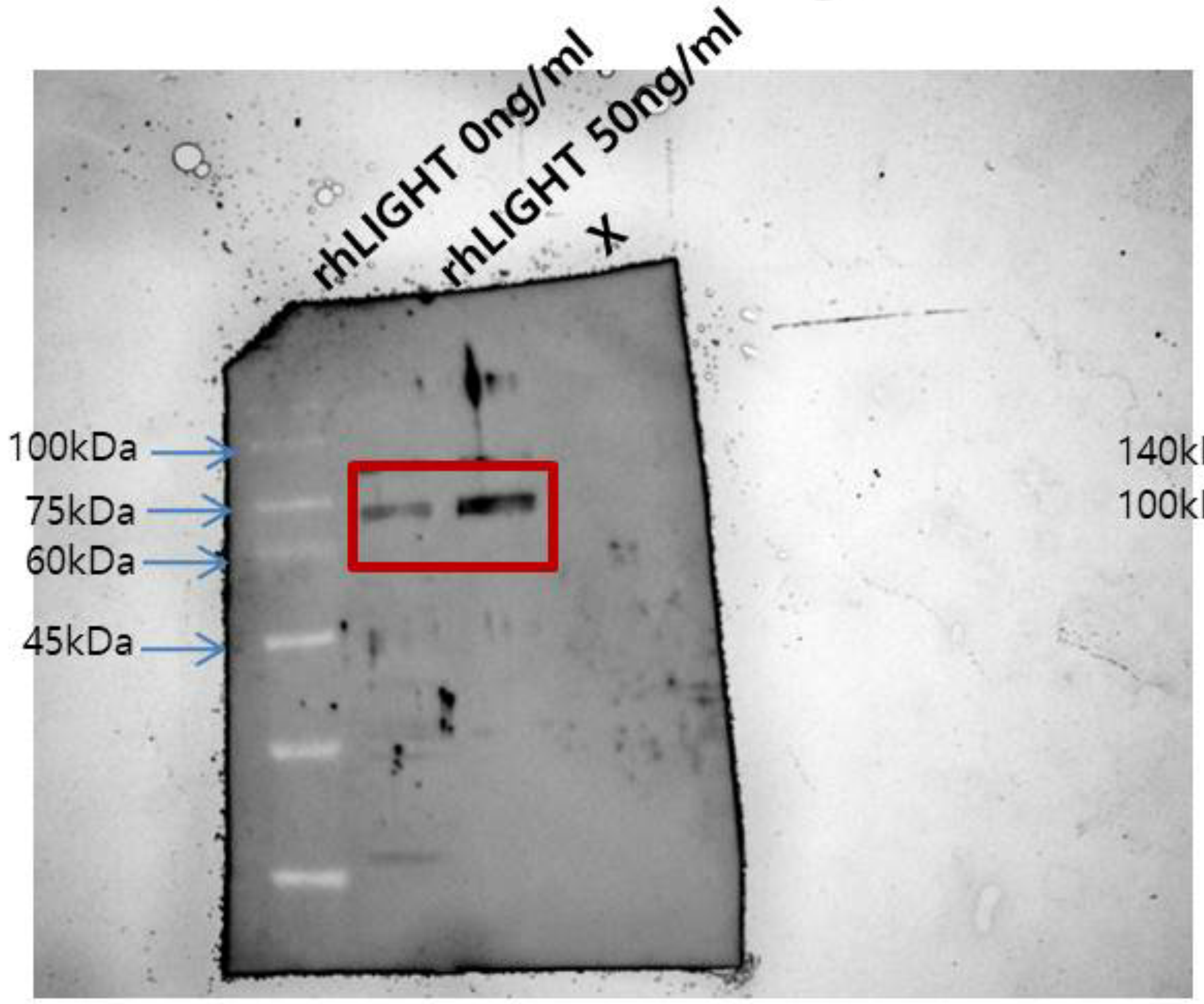

COL1A1/rhLIGHT 0, 50ng/ml

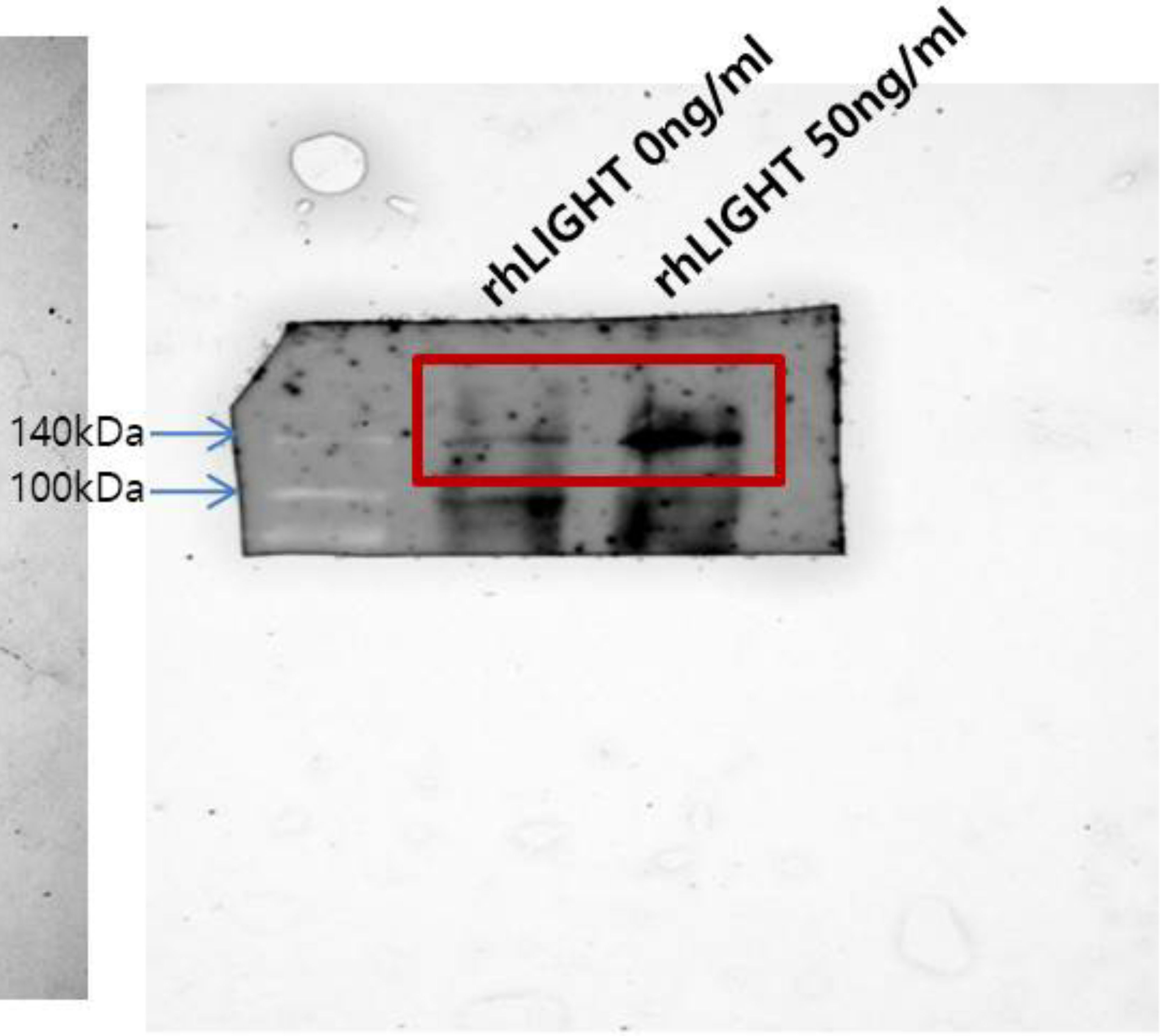

OSX/rhLIGHT 0, 50ng/ml

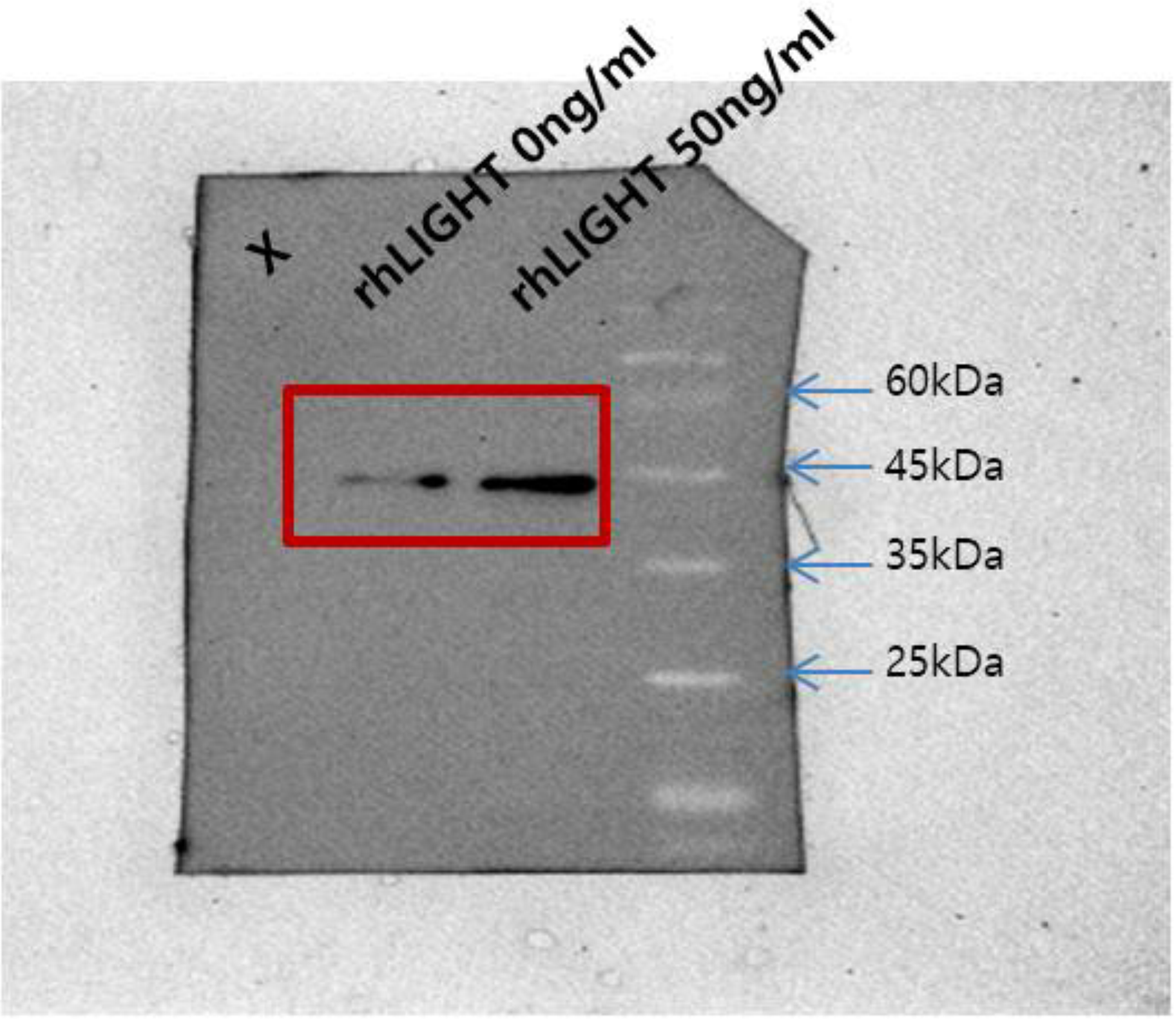

β-actin/rhLIGHT 0, 50ng/ml

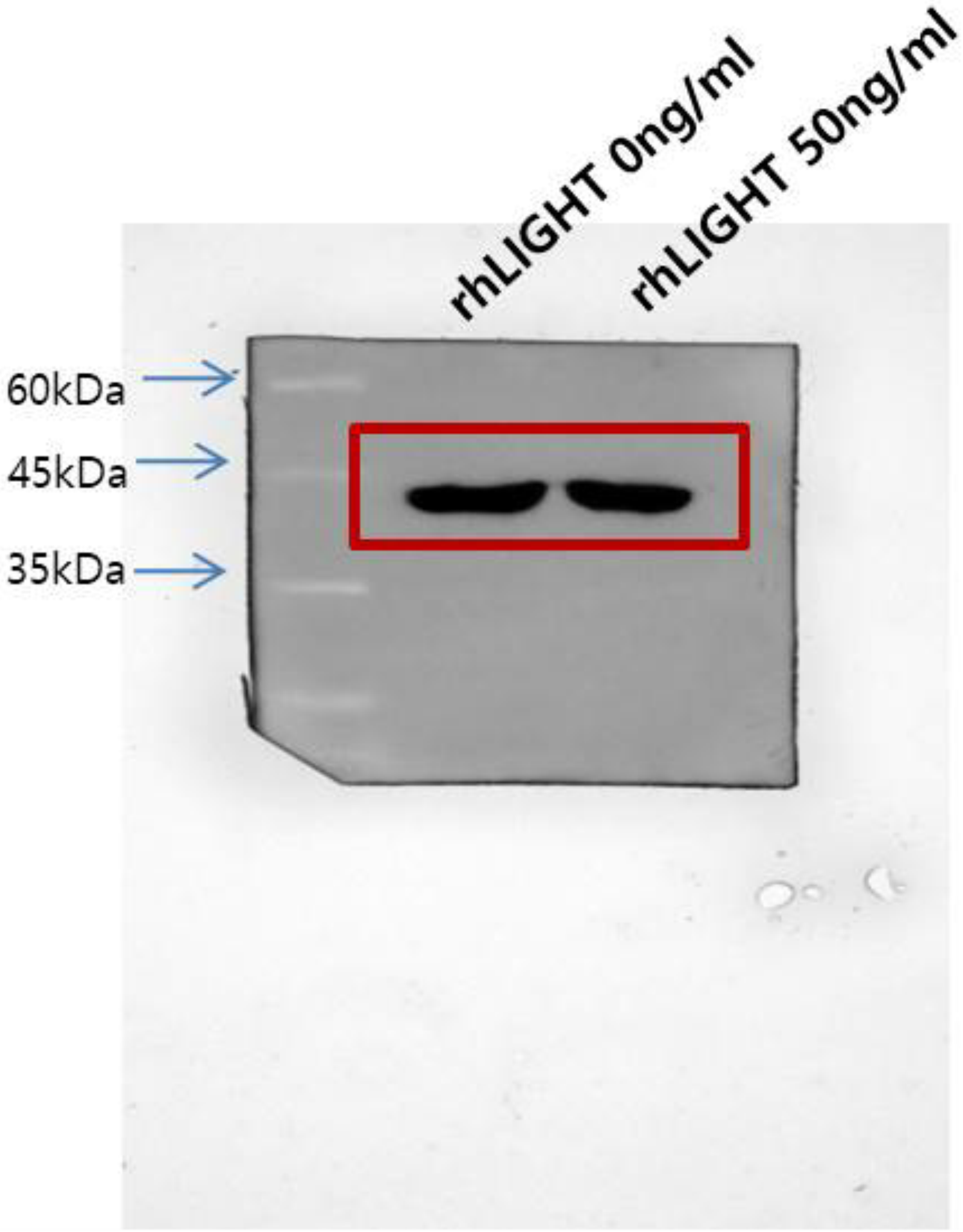

Supplementary Figure 3.
Original western blots used for Fig. 4A

Total β-catenin/ rhLIGHT0, 25, 50ng/ml

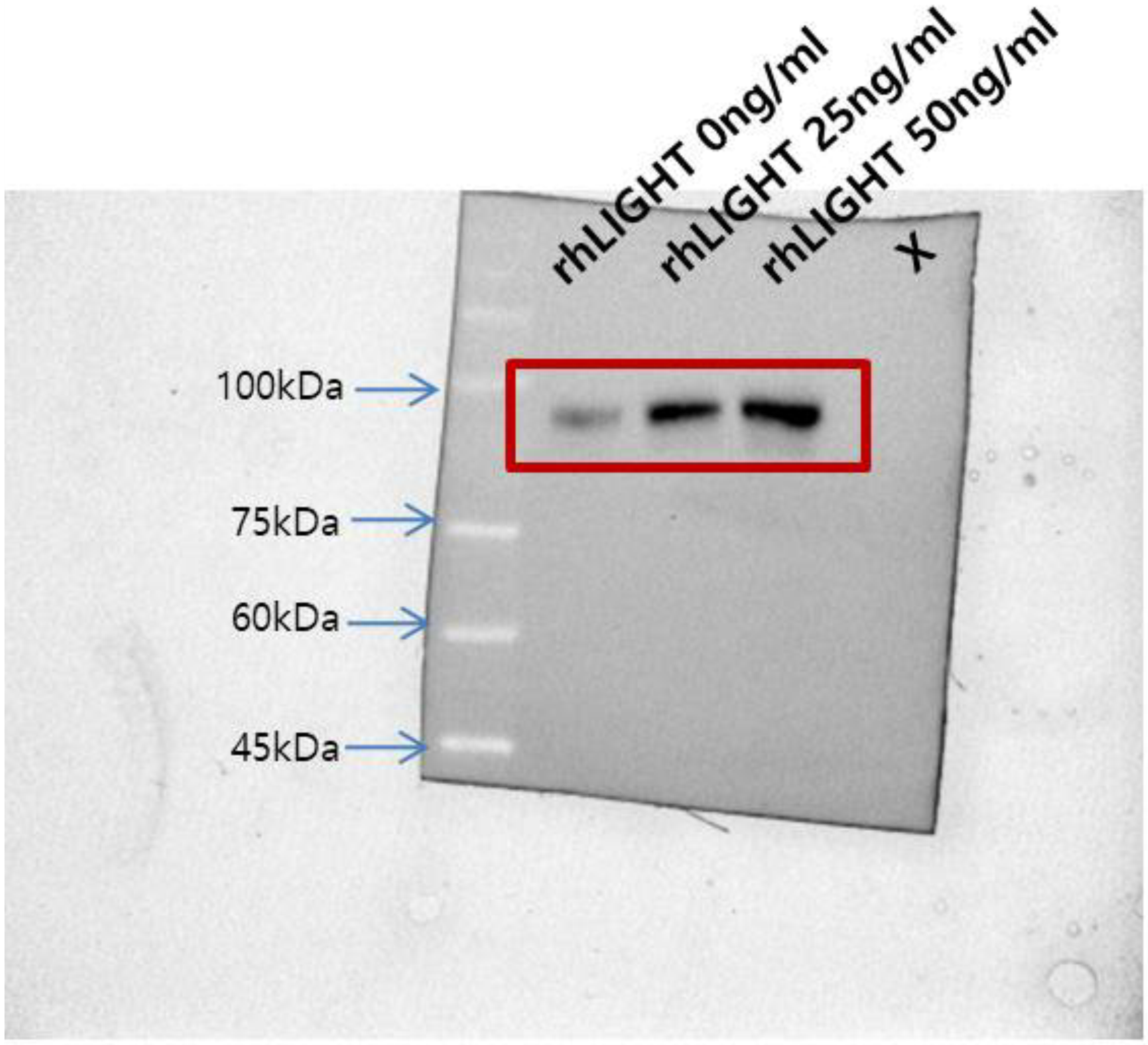

Active β-catenin/ rhLIGHT0, 25, 50ng/ml

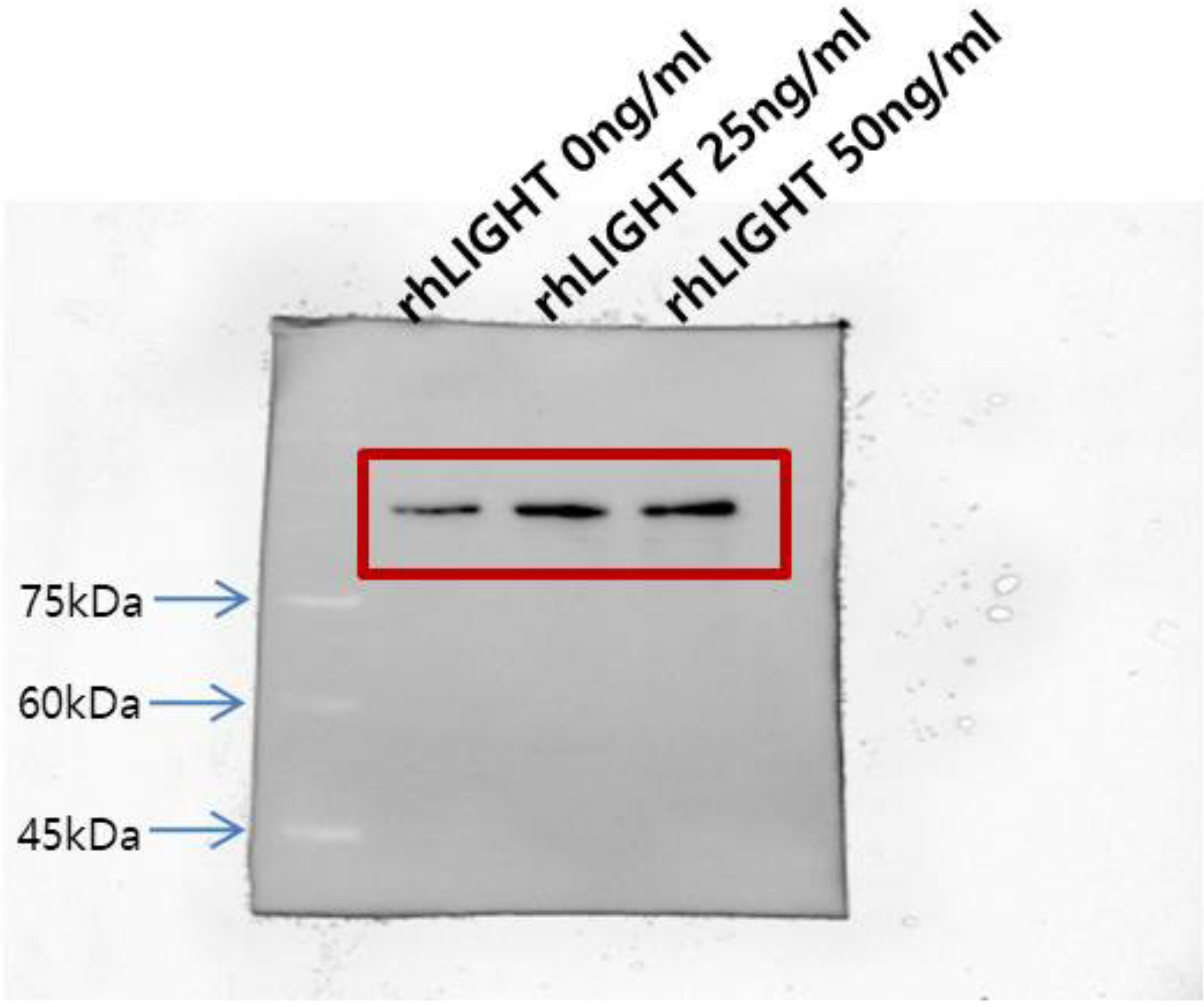

β-actin/rhLIGHT 0, 25,50ng/ml

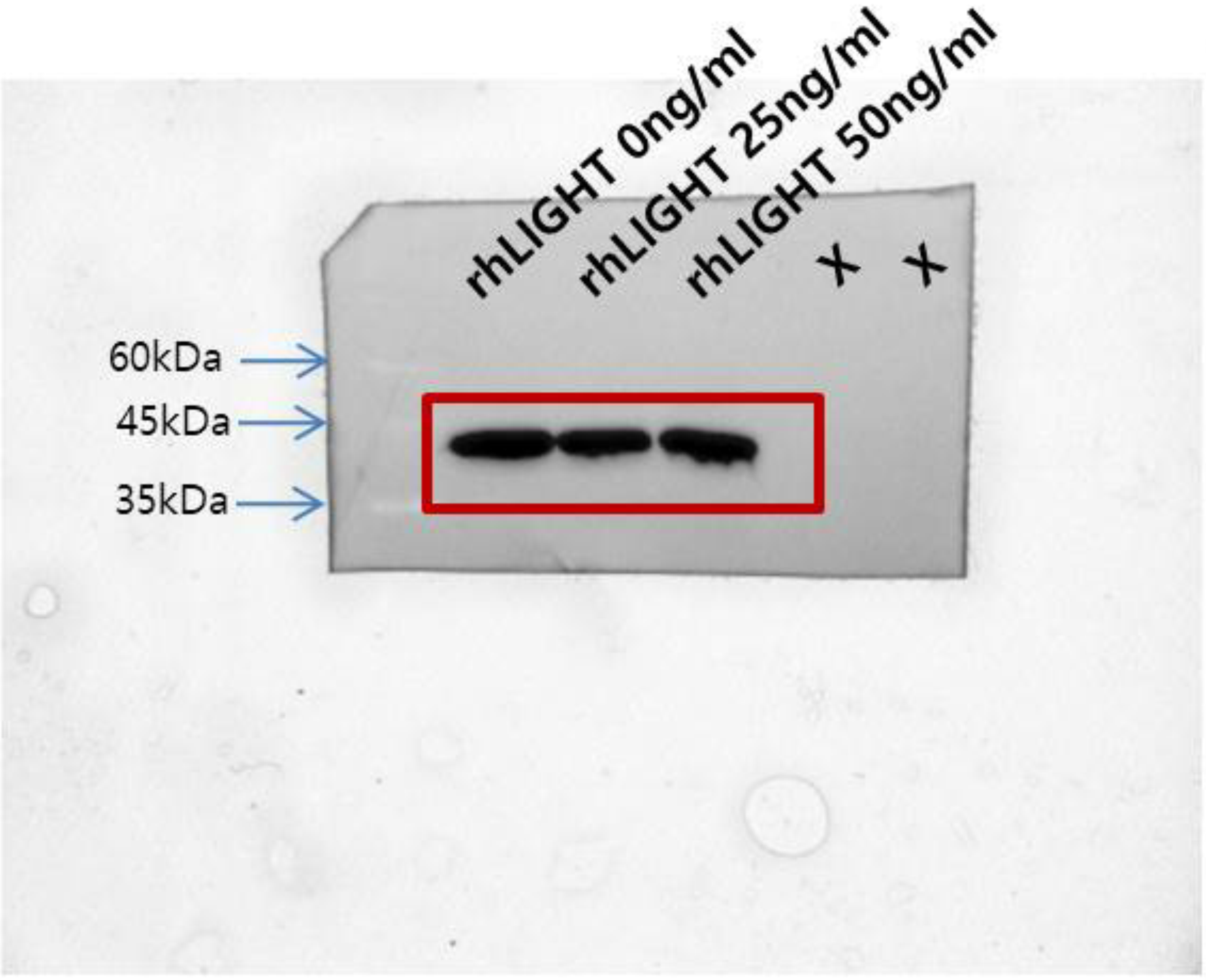

p-ERK/rhLIGHT 0, 25,50ng/ml

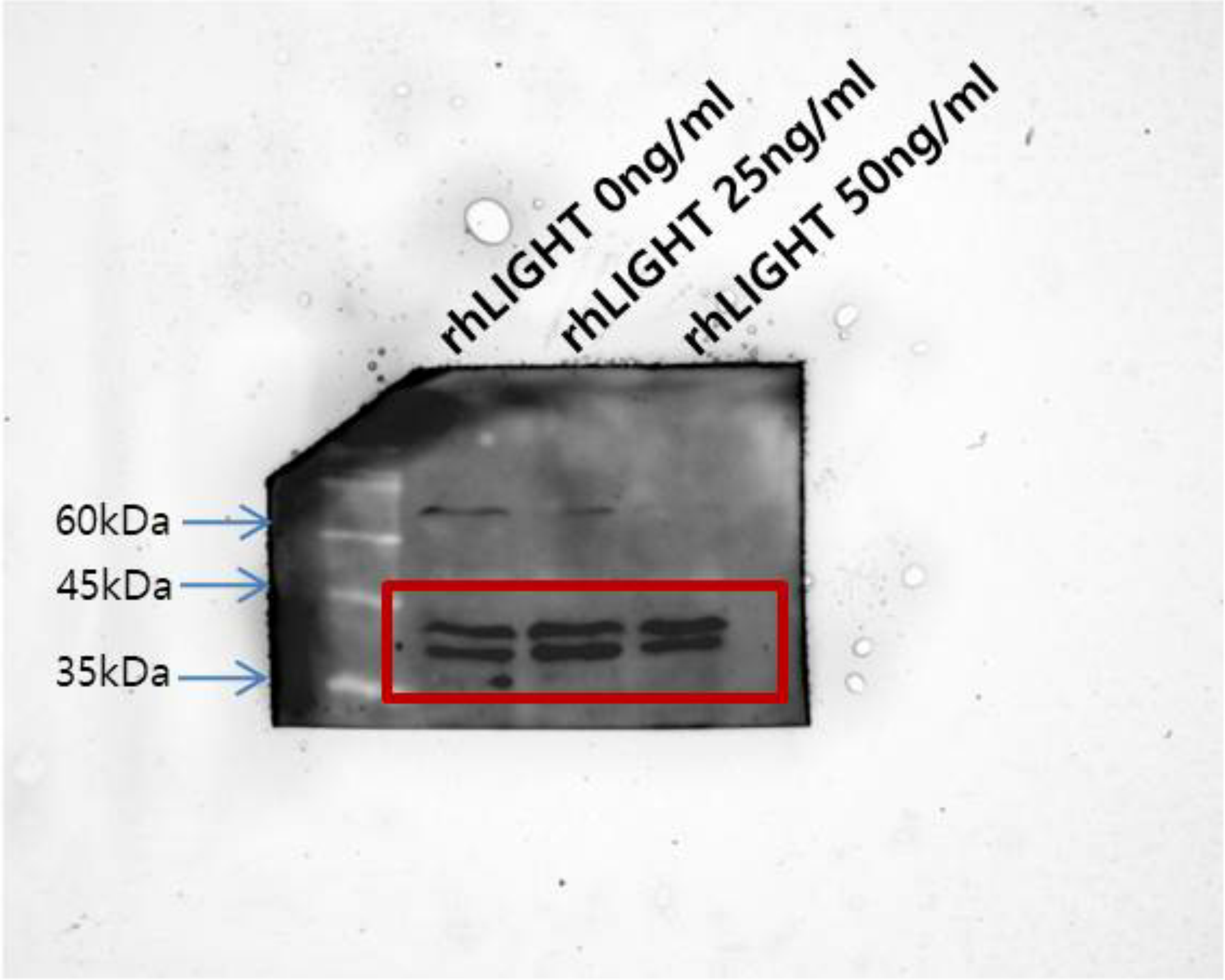

Supplementary Figure 3. continued

Original western blots used for Fig. 4A

ERK/rhLIGHT 0, 25,50ng/ml

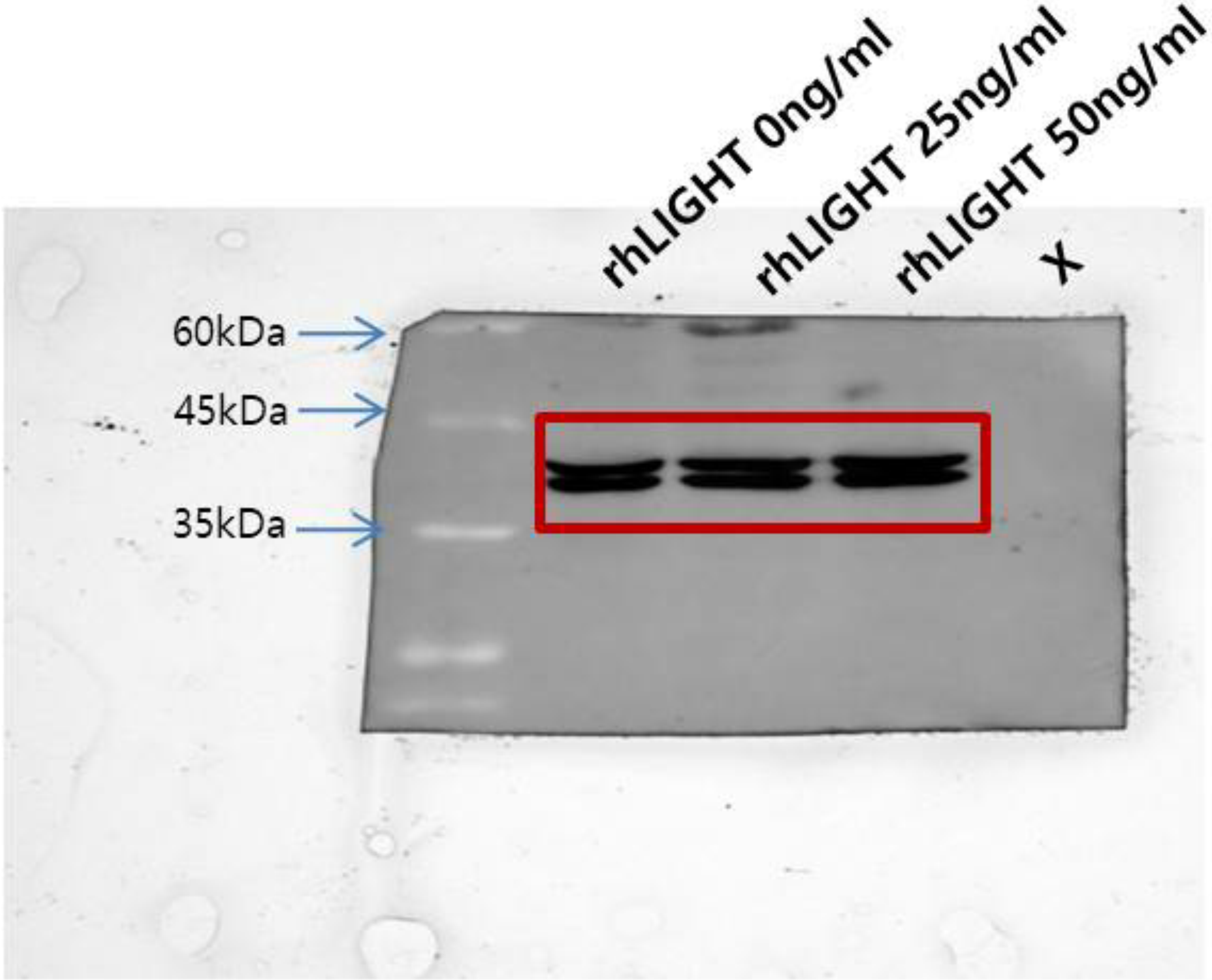

p-p38/rhLIGHT 0, 25,50ng/ml

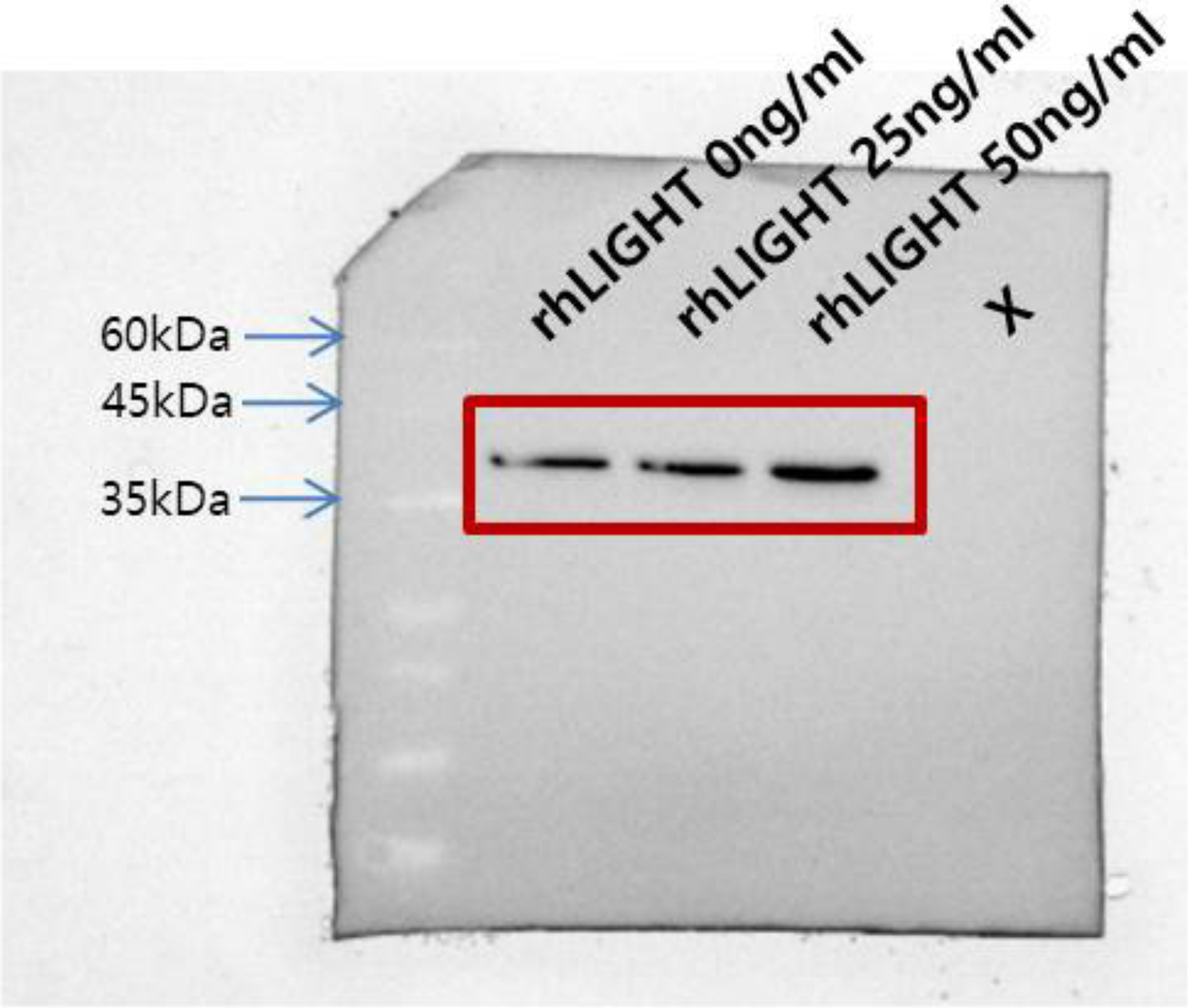

Supplementary Figure 3. continued
Original western blots used for Fig. 4A

p38/rhLIGHT 0, 25,50ng/ml

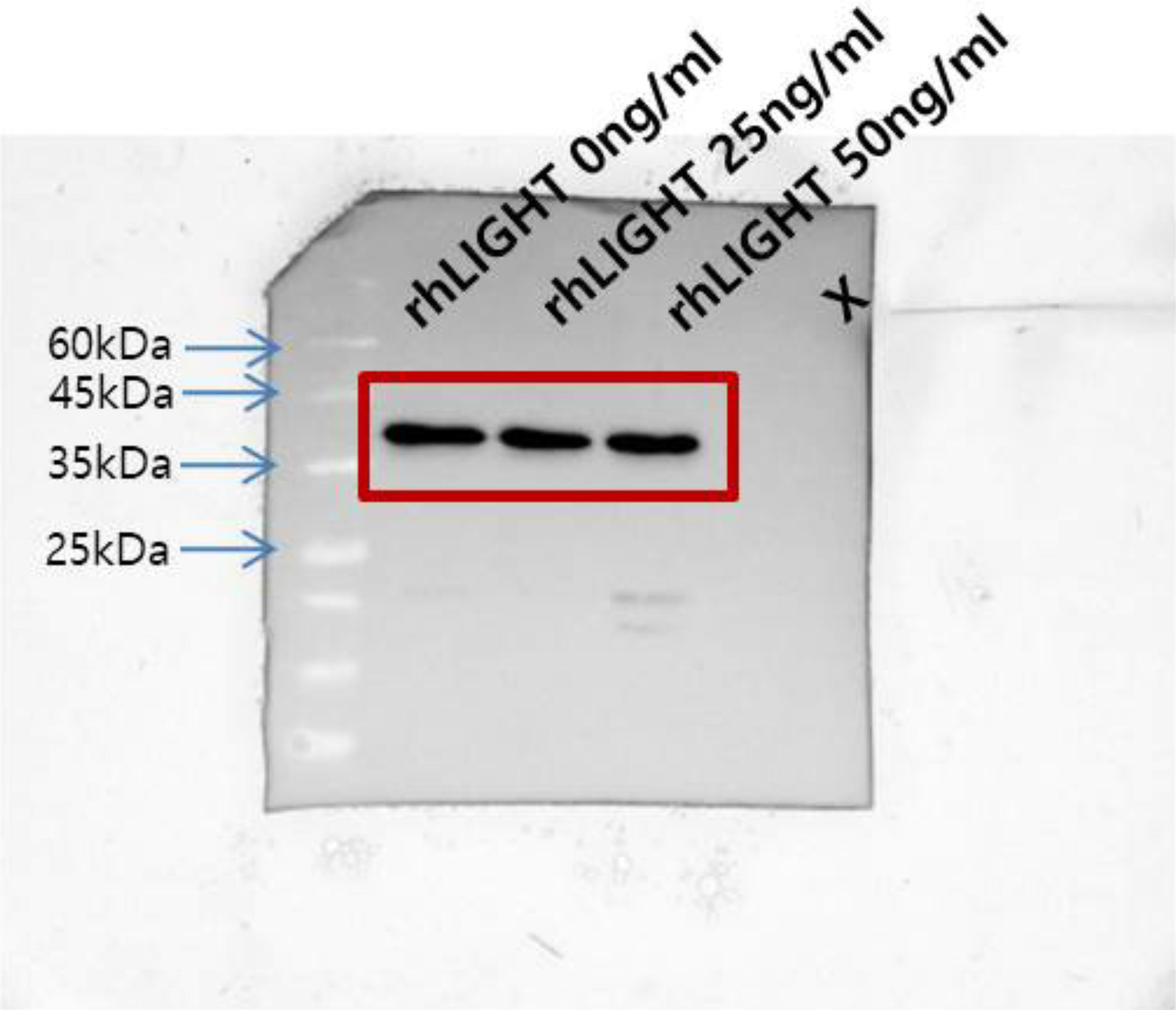

p-JNK/rhLIGHT 0, 25,50ng/ml

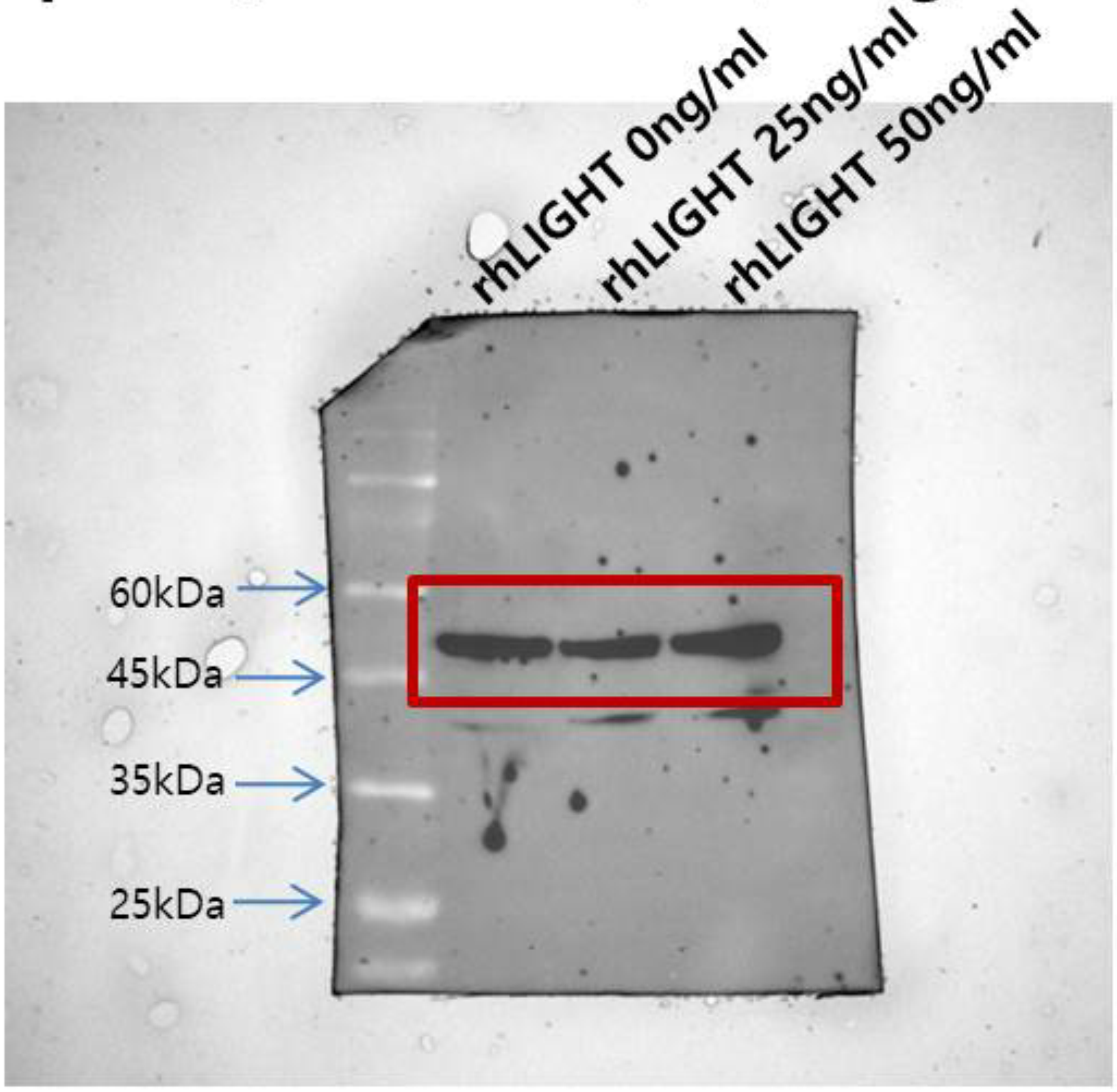

JNK/rhLIGHT 0, 25,50ng/ml

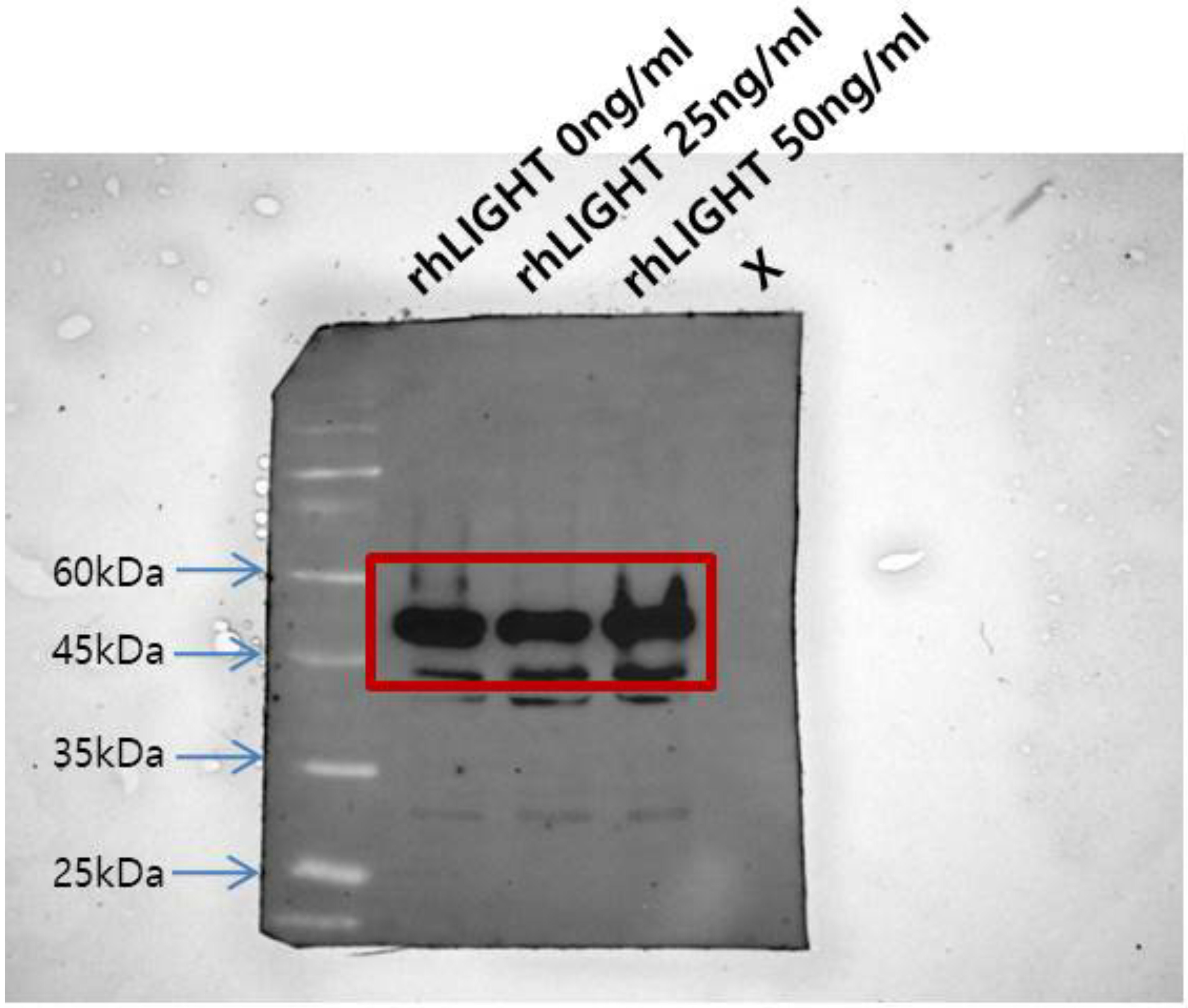

β-actin/rhLIGHT 0, 25, 50ng/ml

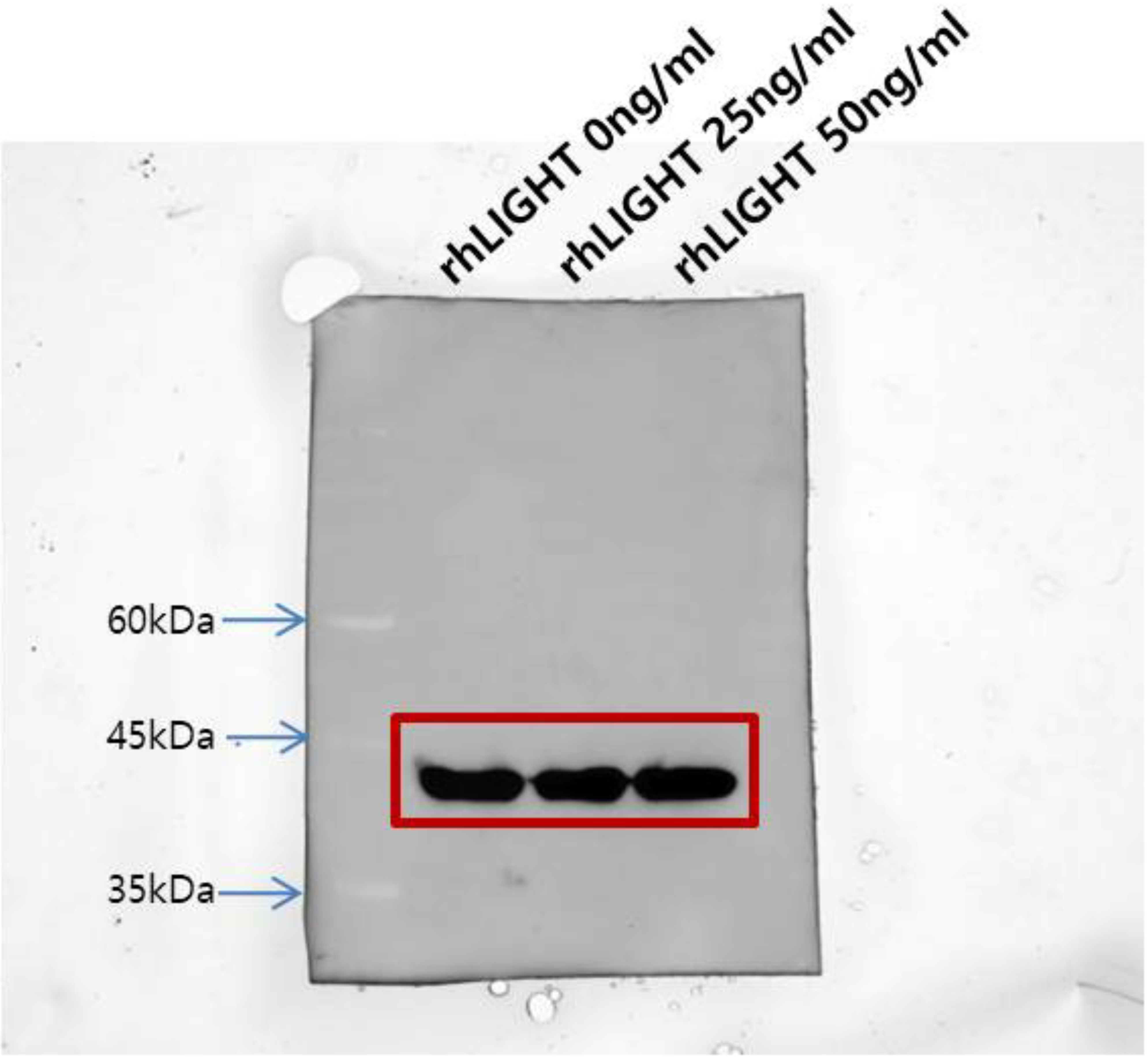

Supplementary Figure 4.
Original western blots used for Fig. 5B

LTβR/rhLIGHT 0, 25, 50ng/ml

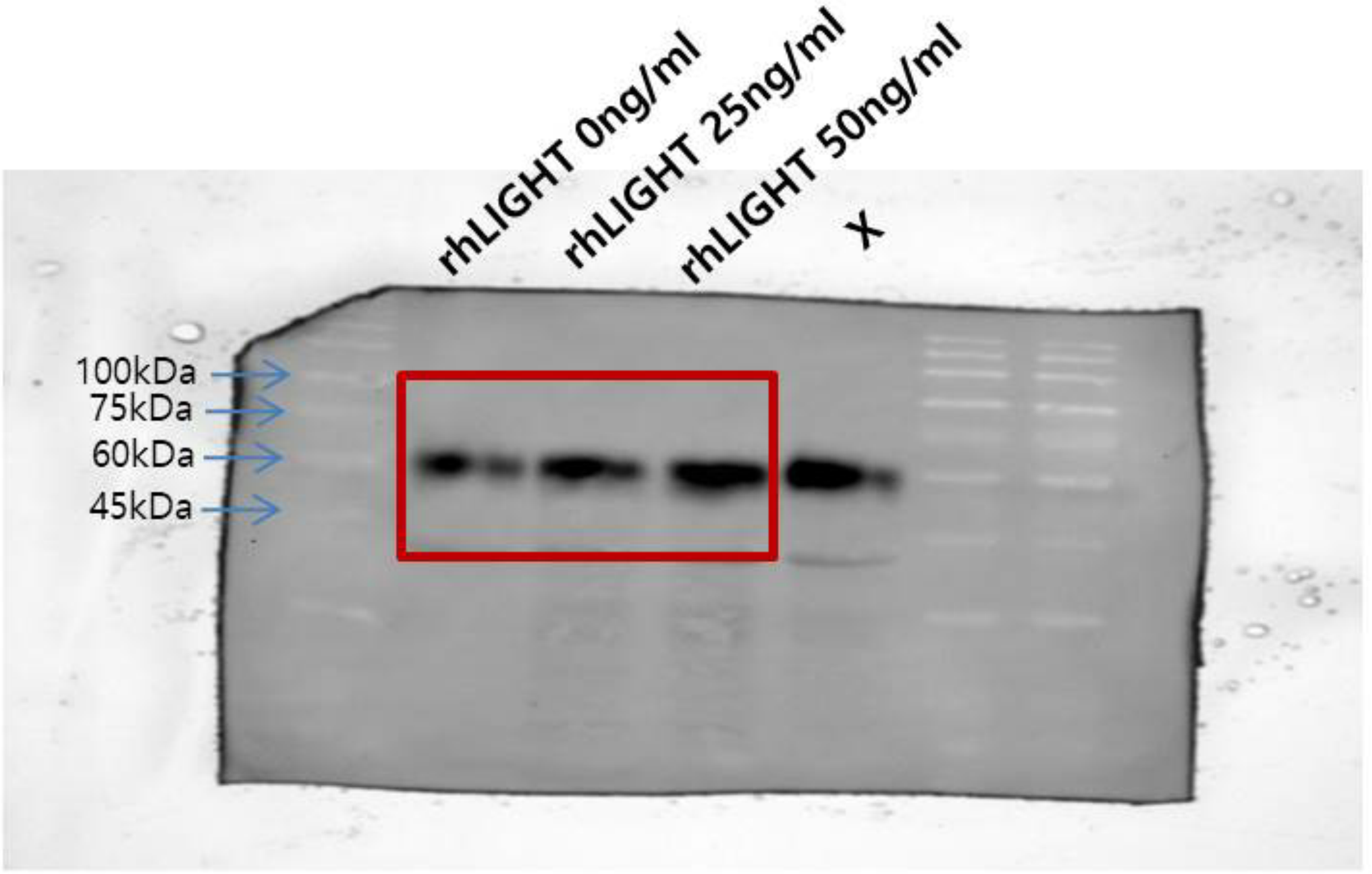

β-actin/rhLIGHT 0, 25, 50ng/ml

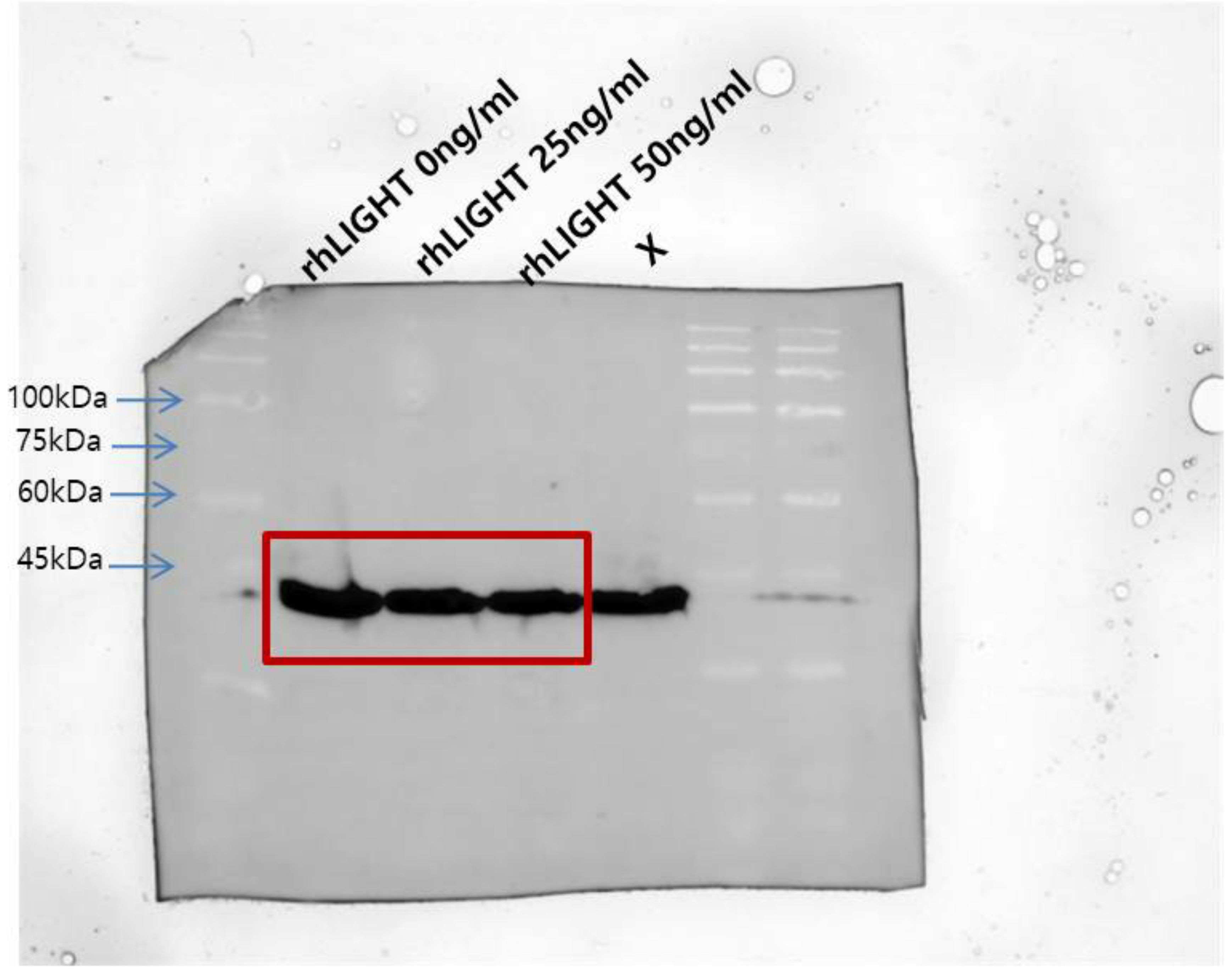

Supplement: S1 Raw images — (PDF) [file pone.0247368.s002.pdf]
